# Non-linear multivariate decomposition modelling of the predictors of menstrual product use among reproductive-aged women: evidence from the 2022 Ghanaian demographic and health survey

**DOI:** 10.3389/frph.2026.1874033

**Published:** 2026-07-07

**Authors:** Benjamin Ansah Dortey, Yula Salifu, Emefa Judith Modey Amoah, Emmanuel Anongeba Anaba, Joseph Lasong, Amidu Alhassan, Adom Manu

**Affiliations:** 1 Winneba Trauma and Specialist Hospital, Winneba, Ghana; 2 Department of Population, Family and Reproductive Health, School of Public Health, University of Ghana, Accra, Ghana; 3 Department of Population, Family and Reproductive Health, School of Public Health, C.K.T. University of Technology and Applied Sciences, Accra, Ghana; 4 Department of Population and Reproductive Health, School of Public Health, University for Development Studies, Tamale, Ghana; 5 School of Nursing and Midwifery, School of Nursing and Midwifery, University of Cape Coast, Cape Coast, Ghana

**Keywords:** decomposition, determinants, Ghana, inequalities, menstrual health, multilevel analysis, policy

## Abstract

**Background:**

Menstrual health is a critical yet under-prioritized component of women's sexual and reproductive health, with persistent inequities in access to menstrual products across socioeconomic and geographic contexts. In Ghana, these disparities are influenced by a combination of individual, household, and community-level factors. This study examined the determinants of menstrual product use and quantified the relative contributions of compositional and structural factors to residence-related disparities using nationally representative data.

**Methods:**

Data were derived from the 2022 Ghana Demographic and Health Survey among a weighted sample of 12,497 women aged 15–49 years. Single-and multilevel logistic and ordered logistic regression models were fitted to identify determinants of menstrual product use, adjusting for intra-cluster correlations and complex survey design. Predictive performance and model fit were evaluated using AIC/BIC, McFadden's pseudo-R², C-index, and ROC AUC. Non-linear multivariate decomposition analysis was conducted to disentangle the contributions of compositional differences (endowments) and differential effects (coefficients) to disparities by urban–rural residence.

**Results:**

Use of modern menstrual products was strongly associated with higher individual educational attainment, household wealth, urban residence, younger age, self-reported good health, and greater media exposure. At the community level, higher average education and media exposure within clusters were also positively associated with uptake. Multilevel modelling revealed that 13%–15% of the variance in product use was attributable to between-cluster differences, highlighting the influence of contextual factors. Predictive models exhibited excellent discrimination (C-index ≥ 0.82; ROC AUC = 0.87). Decomposition analyses indicated that compositional factors, particularly household wealth and education, accounted for the majority of rural–urban (63%) inequalities, while differences in the effects of these determinants further contributed to disparities.

**Conclusions:**

Structural inequities in wealth, education, and community resources are major determinants of menstrual product use in Ghana. Policy interventions that subsidize menstrual products, expand school-and community-based distribution programs, enhance mass media outreach, and improve rural WASH infrastructure are critical to reducing disparities. Integrating menstrual health into national health and gender equity strategies is essential to advancing Sustainable Development Goals related to health, education, and gender equality.

## Introduction

Menstrual health is integral to dignity, bodily autonomy, and participation in education and work, yet large inequities persist in access to appropriate products and facilities. Globally, on any given day, more than 300 million women are menstruating (1). Further, an estimated 500 million lack adequate menstrual products and the enabling environment to manage menstruation safely and privately (1). Recent monitoring by the World Health Organization and the United Nations Children's Fund highlights structural deficits, with fewer than one in three schools globally having bins for menstrual waste in girls' toilets, falling to about one in ten in sub-Saharan Africa, and only two in five schools provide menstrual health education (2). These deficits, alongside affordability constraints and stigma, underscore menstrual health as a public health concern with implications for equity, learning, and productivity (1, 2).

Global and regional evidence indicates substantial variation in product choice and coverage among women of reproductive age. A multi-country analysis across Africa documented a sizable unmet need for menstrual hygiene management (MHM) associated with rural residence, poverty, and limited education (3). In schools, provision of materials and disposal options remains sparse in the region, with only around 11% of schools in sub-Saharan Africa having bins for menstrual waste (2). In Ghana, the 2022 Demographic and Health Survey reports that among women aged 15–49 who menstruated in the preceding year, 88% used disposable sanitary pads and 9% used cloth, indicating near-universal material use but with important heterogeneity in product type that likely reflects cost, availability, culture, and WASH conditions (4). In addition to individual-level factors, community-level drivers such as community education, community media exposure, and community wealth are strongly associated with menstrual practices and may shape awareness, affordability, and access to products (5–7). These factors make menstrual product use among reproductive-aged women a salient health systems and social policy issue.

Despite increasing recognition of menstrual health within Sustainable Development Goal (SDG) target 6.2, equitable access to sanitation and hygiene with attention to the needs of women and girls, two interlinked gaps limit practice, policy, and research. First, national statistics often describe prevalence but rarely decompose disparities to quantify how much of the inequality in product use is attributable to compositional differences vs. differential returns to these characteristics. Without such attribution, programmes risk generic targeting and inefficient resource allocation (8). Secondly, few studies in West Africa integrate decomposition with predictive modelling to identify high-risk profiles for suboptimal product use, limiting proactive service delivery and social protection responses; where decomposition methods have been applied to health outcomes, service delivery, and utilization, they show meaningful, policy-actionable contributions of socioeconomic and community-level factors to gaps (9, 10). Collectively, these gaps perpetuate inequities in access, entrench stigma, and hinder progress towards SDG 6.2 by obscuring where, for whom, and why disparities persist. This study applied non-linear multivariate decomposition to quantify the contributions of socioeconomic, demographic, and community-level factors, including education, media exposure, and wealth, alongside WASH-related determinants to observed gaps in menstrual product use among reproductive-aged women. It also develops predictive models to stratify risk and inform targeted interventions. Evidence from this analysis will support ministries, local governments, and partners to refine subsidies, social marketing, school- and community-based provision, and waste management, while guiding researchers toward indicators that capture not only product uptake but also the enabling environment. In linking decomposition findings to risk prediction, the study will provide actionable intelligence for prioritising resources and monitoring equity-focused progress towards SDG 6.2 in Ghana and comparable African contexts (2, 4).

Although national estimates indicate that most menstruating women in Ghana use some commercial material, these aggregate figures mask differences in the type of product used that carry distinct health, economic, and environmental implications. Continued reliance on traditional materials or nothing among the poorest, alongside near-total dependence on disposable rather than reusable products, signals residual unmet menstrual need that prevalence statistics alone obscure, so characterising who uses which product type identifies the sub-populations that remain underserved. Integrating decomposition with predictive modelling offers complementary, policy-actionable evidence that neither method provides alone: the decomposition attributes the rural–urban gap to its compositional (endowment) and structural (coefficient) sources, indicating where to intervene, while the predictive models identify the individual and contextual profiles most likely to experience suboptimal product use, indicating whom to prioritise.

## Methods

### Study design and source of data

The study analysed data from the 2022 Demographic and Health Survey (2022-GDHS). The data was downloaded from the DHS program website after written permission. The 2022-GDHS was implemented by the Ghana Statistical Service with support from ICF from 17 October 2022 to 14 January 2023. The survey aimed to provide comprehensive data on key demographic and health indicators such as reproductive health, including menstrual health and hygiene, WASH, family planning, and women's empowerment. This information is important to inform policymaking, program evaluation, and the monitoring of health and demographic trends in Ghana.

### Population and sampling

The target population for the women's section included women of reproductive age, thus, aged 15–49. The survey employed a stratified two-stage cluster sampling approach. The 2021 Population and Housing Census list was used as the sampling frame. Enumeration areas (clusters) were selected in the first stage, and households were selected in the second stage. 618 clusters were selected from the sampling frame using probability proportional to size for urban and rural locations in each of the 16 regions in the country. In the second stage, the household lists were updated through household listings in each of the selected clusters. The updated list served as a sampling frame for the random selection of households. The survey randomly selected a fixed number of 30 households from each cluster. A detailed sampling procedure is available at the DHS program website (https://www.dhsprogram.com). This rigorous design ensures representation at both national and sub-national levels. The 2022-GDHS successfully interviewed 15,014 women aged 15–49 years (98% response rate). This study focused on women aged 15–49 years with complete data on menstrual hygiene/health parameters. Therefore, a total weighted sample of 12,497 reproductive-aged women aged 15–49 years was included in this analysis.

### Definition of study variables

The outcomes of this study were modern menstrual product use (binary: yes/no) and general menstrual product use (ordered: nothing/other, traditional and modern).

However, the covariates adjusted for were at the individual, household, and community levels. The community level covariates were region, community education and community mass media exposure, and community wealth status. Also, the household-level covariates were household wealth status, household mass media exposure, place of residence, and household WASH status. The individual-level covariates were age, level of education, religion, self-reported health status, age at menarche, and marital status.

### Measurements of variables

#### Outcome measure

The dependent variable was menstrual product use, derived from DHS variables on materials used to manage menstruation. Responses were grouped into three mutually exclusive categories: *nothing/other* (women who reported using underwear only, unconventional materials, or nothing), *traditional* (cloth, toilet paper, cotton wool, or other non-commercial absorbents), and *modern* (disposable sanitary pads, reusable pads, tampons, or menstrual cups). Among those classified as modern users, a secondary binary outcome was generated to distinguish between *sustainable products* (reusable pads and menstrual cups) and *disposable products* (commercial disposable pads and tampons). For analytical purposes, a new variable was generated (modern menstrual product use) and coded as modern use vs. others (traditional, nothing/others).

#### Water, sanitation, and hygiene (WASH)

Household WASH conditions were measured using three DHS indicators. WASH classifications were defined per the WHO/UNICEF Joint Monitoring Programme (JMP) standards. First, the *source of drinking water* was coded as *improved* if the household reported piped water, boreholes, protected wells, protected springs, rainwater, bottled water, or sachet water, and as *unimproved* otherwise. Second, a *sanitation facility* was considered *improved* if the household reported flush toilets to sewer or septic, ventilated improved pit (VIP) latrines, pit latrines with slabs, or composting toilets; open defecation, pit latrines without slabs, and bucket or hanging toilets were classified as *unimproved*. Third, the *time to the water source* was coded as *≤*30 min *round trip* (including water on premises) (improved) vs. *>*30 min *(unimproved)*. A binary composite WASH index classified households as *improved* only if all three components were improved.

#### Covariates

Marital status was re-coded into *never married*, *currently married/cohabiting*, and *formerly married* (widowed, separated, or divorced). Self-reported health status was also re-coded, with original categories collapsed into three groups: *good/very good*, *moderate*, and *poor/very poor*. Mass media exposure was constructed from reported frequency of exposure to newspapers, radio, and television; the highest reported frequency was used to classify respondents as *no exposure*, *less than once a week*, or *at least once a week*. Age at menarche was recategorized into early menarche (<12 years), normal menarche (12–15 years), and late menarche (16 or more years).

Household wealth status was measured using the standard DHS wealth index, an asset-based measure constructed through principal components analysis of household assets, dwelling characteristics, and access to services, which is partitioned into five quintiles ranging from poorest to richest.

### Analysis of data and management

#### Handling missingness

Data were assessed for missingness, and incomplete or missing data on menstrual hygiene were dropped. All missing data relevant to the analysis were deleted using the list-wise deletion method. Missing data were less than 5%. All analysis and cleaning were conducted in STATA 18/MP.

#### Descriptive analysis

Descriptive analysis was conducted to summarize participant characteristics and presented as frequencies, percentages, and proportions with their corresponding 95% confidence intervals.

#### Multivariable analysis

Single-level multivariable and multi-level multivariable analysis were conducted. The binary logistic regression and ordered regression model were fitted for binomial [modern menstrual product use (yes/no)] and ordered outcomes [menstrual product use (no/others, traditional, modern)], respectively, to assess the predictors of menstrual product use. Model parameters were obtained using maximum likelihood estimations. The multi-level model adjusted for intra-cluster correlations and estimated the random intercept effect of the models. However, the single-level model did not focus on between-group and within-group comparisons. Also, the single-level modelling approaches did not model the community-level random effects and treated community-level variables as fixed. To quantify the extent of community-level variation and assess the improvement in model fit, we compared multilevel models to their single-level counterparts. All covariates were simultaneously adjusted for in both single and multi-level models. The results were presented as adjusted odds ratios (AORs) with corresponding 95%confidence intervals. A 5% significance level was set for all multivariable estimations.

#### Random intercept estimations

The inter-cluster correlation (ICC) was estimated to measure the proportion of total variance in the use of menstrual products that is explained by clustering. For ICC close to 0, most variability is at the individual level, and the clustering effect is weak, while for ICC closer to 1, there is a strong clustering effect, in that outcomes depend heavily on which cluster people belong to. Also, median odds ratios (MORs) were estimated to measure heterogeneity in the multilevel regression models. However, MOR close to 1 indicated little cluster heterogeneity, MOR ≥ 1.5 indicated moderate cluster effect, and MOR ≥ 2 indicated strong heterogeneity. PSU Variance (*τ*²) was further estimated to quantify between-cluster variability of the models.

#### Model fitness and multicollinearity assessment

Goodness of fit was assessed using the likelihood ratio test. Also, in both the single-level and multi-level analyses, Akaike and Bayesian Information Criteria (AIC/BIC) estimates were used to assess model fitness. Lower information criteria indicated a better fit. Moreover, McFadden Pseudo *R*-square was estimated for both models to also assess the fitness of the models, and values of 0.2 and beyond indicate good model fit. To check multicollinearity, the generalised variance-inflation factor (GVIF) was used, and a GVIF value less than 10 was considered acceptable. However, GVIF was less than 5.

#### Predictive model estimations and assessment

The Somers' D and Concordance index (C-index) was used to assess the predictability of the ordered regression models. This is often the most efficient and interpretable single metric for ordinal outcomes, as it generalizes the Area Under Curve (AUC) perfectly. The C-index represents the probability that, given two randomly selected individuals, the model will assign a higher predicted risk or score to the individual with the worse actual outcome, as done in AUC; AUC = 0.5 represents model predictions that are no better than random guessing, and AUC = 1.0 represents perfect discrimination for all pairs.

Thus, the Somers' D and C-index evaluate all possible pairs of observations across all ordinal levels and does not require arbitrarily choosing a cutoff. In this estimation, a rank-based statistic (rank correlation measure) is estimated after an ordered logistic model. The “*somersd”* command was used in this estimation. The C-index is calculated as (Somers' D + 1)/2, which is directly analogous to the AUC. Somers' D value ranges from −1 to 1, where 1 indicates perfect concordance. The C-index ranges from 0.5 to 1.0, where: 0.5 = No predictive ability (random guessing)**,** 0.7–0.8 = Acceptable discrimination, 0.8–0.9 = Excellent discrimination, and 0.9 = Outstanding discrimination. After this estimation, the distribution of predicted values by outcome category was examined using a kernel density plot to visualize model discrimination. For binary regression models, the Receiver Operating Characteristic (ROC) Curve was used to estimate the predictability of the models. An AUC of 1.0 indicated perfect discrimination.

#### Community-level variable estimations

Community-level variables were constructed by aggregating individual-level data from the DHS to the Primary Sampling Unit (PSU) level, which serves as a proxy for the local community. The mean value of each characteristic was calculated for all women within each PSUs. This approach captured the compositional effect or the overall socio-economic environment of the community.

Three community-level variables were created:

1. Community Education: The mean years of education among women within the PSU.
2. Community Media Exposure: The mean frequency of mass media exposure (reading newspapers, listening to the radio, watching television) among women within the PSU.
3. Community Wealth: The mean wealth index (based on the DHS wealth quintile) of households within the PSU.

To enhance the interpretability of the regression coefficients and reduce multicollinearity, these community-level measures were grand-mean centred. This transformation represented each community's value as a deviation from the national mean. Consequently, the coefficient for a community-level variable in the model represented the change in the outcome (menstrual product use) associated with living in a community that is one unit above the national average for that characteristic, all else being equal. The distributions of these aggregated variables were assessed for normality using visual inspection of quantile-quantile plots before analysis.

#### Decomposition analysis

A non-linear multivariate logistic decomposition analysis was conducted to understand the inequities in modern menstrual product use. The “*mvdcmp”* and “*svylogit”* prompts were used in these estimations. The decomposition was conducted by place of residence to understand the urban-rural inequalities associated with menstrual hygiene practices.

This approach moves beyond identifying correlates of menstrual product use and quantifies the extent to which observed differences across population groups (e.g., rural–urban) are attributable to differences in characteristics (endowments) vs. differences in the effects of those characteristics (coefficients).

The non-linear decomposition followed the Powers–Yun extension of the Blinder–Oaxaca approach for models with a non-linear link.

For the logistic model, the difference in the mean predicted probability of modern product use between rural (A) and urban (B) women was partitioned as follows:

$${\bar{Y}}_{A}-{\bar{Y}}_{B}=[\underset{\mathrm{Total}\;\mathrm{gap}}{\underbrace{\bar{F}(X_{A}\beta_{A})-\bar{F}(X_{B}\beta_{B})}}]=[\underset{\mathrm{Endowment}\;\left(E\right)}{\underbrace{\bar{F}(X_{A}\beta_{A})-\bar{F}(X_{B}\beta_{A})}}]+[\underset{\mathrm{Coefficient}\;\left(C\right)}{\underbrace{\bar{F}(X_{B}\beta_{A})-\bar{F}(X_{B}\beta_{B})}}]$$

where *F* is the logistic function, *X* denotes the covariate means, and *β* the estimated coefficients for each group. The first bracketed term is the endowment (characteristics, E) component, capturing the portion of the gap attributable to group differences in the distribution of covariates; the second is the coefficient (returns, C) component, capturing the portion attributable to differences in the estimated effects of those covariates, including the intercept. A positive detailed contribution indicates that a variable widens the rural–urban gap, whereas a negative contribution narrows it. The decomposition assumes correctly specified models and comparable measurement of covariates across groups; the choice of reference group and the scaling of the coefficient component require caution, and, as with any decomposition of observational data, the components describe statistical sources of the gap rather than causal effects.

### Survey weighting and adjustments

The analysis accounted for sampling weights, clustering, and stratification to reflect the complex survey design methodology. This is important to obtain accurate Taylor Linearized estimates. The “*svy*” command was used to adjust for these and set the data to survey.

To incorporate the complex survey design, the data were declared as survey data in STATA using the primary sampling unit (cluster) as the sampling unit, the sample stratum as the strata, and the women's individual sampling weight (v005 divided by 1,000,000) as the probability weight. For the single-level models, design-based standard errors were obtained through Taylor-series linearisation under this specification. For the multilevel models, a two-level random-intercept binary logistic (melogit) and ordered logistic (meologit) specification was fitted with women nested within clusters, with the sampling weights applied as level-one probability weights and the cluster as the level-two random effect, so that both the survey weighting and the hierarchical structure were accommodated simultaneously. Between-cluster variation was summarised using the cluster-level variance (*τ*²), the intraclass correlation coefficient (ICC), and the median odds ratio (MOR), and the fit of the multilevel models was compared with their single-level counterparts using the likelihood-ratio test alongside the AIC and BIC.

### Ethical considerations

The 2022 GDHS protocol was approved by the Ghana Health Service Ethics Review Committee and the ICF Institutional Review Board. Before data collection, informed consent was obtained from all survey respondents, and confidentiality and privacy were prioritized during data collection. A further analysis of the DHS data does not require ethics approval.

## Results

### Sample characteristics

Table 1 presents the weighted distribution of participants and their use of modern menstrual products. Many women were married or cohabiting (49.9%), with unmarried women forming a substantial minority (39.8%). Product use was markedly higher among married women compared to those formerly married. Self-reported health was largely favourable, as over three-quarters described their health as good or very good, among whom nearly four in five used modern products. By contrast, use was minimal among women reporting poor health. Most participants attained menarche between the ages of 12–15 (69.9%), and this group accounted for the largest share of product users. Regarding media exposure, regular household access (≥once weekly) was common (74.1%) and strongly associated with product uptake, whereas those without exposure had the lowest prevalence.

**Table 1:** Sample characteristics.

(A) Participant characteristics (Left panel) [N-weighted = 12,497]			(B) Proportion of modern menstrual product use across characteristics (Right panel)		
Variables	Weighted frequency (*N*)	Weighted percentage (%)	Proportion	95% CI	
Individual marital status					
Never in union	4,971	39.8	0.429	0.415	0.444
Married/living with partner	6,236	49.9	0.472	0.458	0.486
Widowed/Divorced/Separated	1,290	10.3	0.099	0.091	0.108
Individual self-reported health status					
Good/Very Good	9,611	76.9	0.781	0.766	0.794
Moderate	2,497	20.0	0.194	0.182	0.207
Bad/Very Bad	388	3.1	0.025	0.021	0.030
Age at menarche in years					
Early (<12)	497	4.0	0.040	0.035	0.046
Normal (12–15)	8,668	69.9	0.708	0.695	0.721
Late (>15)	3,234	26.1	0.251	0.239	0.264
Household mass media exposure					
No exposure	1,450	11.6	0.095	0.086	0.105
Less than once a week	1,793	14.3	0.143	0.132	0.154
At least once a week	9,254	74.1	0.763	0.748	0.777
Household WASH status					
Unimproved WASH	4,244	34.8	0.312	0.287	0.338
Improved WASH	7,943	65.2	0.688	0.662	0.713
Household wealth status					
Poorest	1,779	14.2	0.116	0.102	0.132
Poorer	2,193	17.6	0.164	0.148	0.181
Middle	2,624	21.0	0.209	0.193	0.227
Richer	2,914	23.3	0.247	0.230	0.266
Richest	2,987	23.9	0.263	0.238	0.289
Individual educational level					
No education	1,749	14.0	0.102	0.093	0.110
Primary	1,602	12.8	0.122	0.113	0.131
Secondary	7,780	62.3	0.655	0.640	0.670
Higher	1,367	10.9	0.122	0.109	0.135
Place of residence					
Urban	7,386	59.1	0.624	0.607	0.641
Rural	5,111	40.9	0.376	0.359	0.393
Region					
Western	814.0	6.5	0.064	0.058	0.071
Central	1,450	11.6	0.116	0.105	0.129
Greater accra	2,004	16.0	0.175	0.158	0.193
Volta	600	4.8	0.046	0.039	0.054
Eastern	1,041	8.3	0.084	0.076	0.094
Ashanti	2,504	20.0	0.206	0.189	0.225
Western north	353	2.8	0.030	0.026	0.034
Ahafo	274	2.2	0.023	0.021	0.026
Bono	484	3.9	0.042	0.037	0.049
Bono east	561	4.5	0.045	0.040	0.051
Oti	305	2.4	0.021	0.018	0.024
Northern	884	7.1	0.052	0.045	0.059
Savannah	245	2.0	0.017	0.013	0.021
Northeast	202	1.6	0.015	0.013	0.017
Upper east	471	3.8	0.040	0.034	0.046
Upper west	306	2.5	0.025	0.021	0.029
Individual age in years					
15–19	2,491	19.9	0.212	0.202	0.221
20–24	2,334	18.7	0.199	0.189	0.210
25–29	1,848	14.8	0.153	0.143	0.162
30–34	1,784	14.3	0.141	0.133	0.151
35–39	1,709	13.7	0.130	0.122	0.139
40–44	1,431	11.4	0.104	0.096	0.112
45–49	900	7.2	0.061	0.054	0.068
Menstrual product use					
Nothing/others	230	1.8			
Traditional	1,274	10.2			
Modern	10,994	88.0			
Modern menstrual product use					
Modern	10,994	88.0			
Traditional/nothing/others	1,504	12.0			
Types of menstrual products used					
Sustainable	269	2.4			
Disposable	11,057	97.6			

WASH, water, sanitation and hygiene; *N*, frequency; %, percentage; 95%CI, 95% confidence interval.

Overall, 88.0% of women used modern menstrual products, 10.2% used traditional materials, and 1.8% used nothing or other materials; among modern users, 97.6% used disposable products and 2.4% used sustainable (reusable) products (Table 1).

Two-thirds of households had improved WASH facilities, and women from such households reported significantly higher use than their counterparts without access. Wealth gradients were also clear; product uptake rose steadily across quintiles, with women in the richest households reporting more than twice the use observed in the poorest. Educational attainment showed a similar gradient. Secondary education, reported by 62.3% of participants, was associated with the highest use, while women with no schooling reported the least. Moreso, urban residence (59.1%) was strongly correlated with use, with urban women reporting nearly two-thirds of total users. Regionally, Greater Accra and Ashanti stood out with higher prevalence, while northern and newly created regions recorded much lower uptake. Finally, age distribution showed that adolescents (15–19 years) constituted the largest age group and were also the most likely to use modern menstrual products, with prevalence declining steadily with increasing age (Table 1).

### Single-level and multi-level ordered logistic regression model of determinants of menstrual product use (nothing/others vs. traditional vs. modern)

We fitted single-level and random-intercept ordered logistic regression models for menstrual product use coded as 0 = no/other, 1 = traditional, 2 = modern (higher category = greater likelihood of using traditional/modern products). Model fit improved when accounting for cluster (PSU) heterogeneity: the single-level model had log-likelihood ll(model) = −4,093.03 and McFadden's R² = 0.1950 (AIC = 8,272.06, BIC = 8,589.23), whereas the multilevel random-intercept model produced ll(model) = −4,000.998, McFadden's R² = 0.2131 (AIC = 8,089.997, BIC = 8,414.546), indicating better fit for the multilevel specification. Further, the random intercept captured substantial between-cluster variation: the estimated cluster variance was *τ*² = 0.505 (95% CI: 0.381–0.670; SE = 0.073), the intraclass correlation coefficient (ICC) was 0.133 (13.3%), and the median odds ratio (MOR) was 1.97. These statistics indicate that roughly 13% of the total variability in the ordered outcome is attributable to differences between clusters and that the median cluster effect nearly doubles the odds of higher product use (Table 2).

**Table 2:** Single-level and multi-level ordered logistic regression model of determinants of menstrual product use.

Single level ordered logistic regression model of determinants of menstrual product use (Left Panel)					Multi-level ordered logistic regression model of determinants of menstrual product use (Right Panel)				
Variables	AOR	*P*-value	95% CI		Variables	AOR	*P*-value	95% CI	
Community mass media exposure	1.175	0.332	0.848	1.627	Community mass media exposure	1.183	0.370	0.820	1.706
Community education	1.593	0.019*	1.080	2.348	Community education	1.761	0.003*	1.212	2.560
Community poverty	0.851	0.140	0.686	1.054	Community poverty	0.830	0.081	0.673	1.023
Individual Marital status					Individual Marital status				
Never in Union	Ref				Never in Union	Ref			
Married/Living with Partner	0.600	<0.001***	0.452	0.797	Married/Living with Partner	0.578	<0.001***	0.434	0.769
Widowed/Divorced/Separated	0.611	0.011*	0.419	0.892	Widowed/Divorced/Separated	0.582	0.006**	0.395	0.857
Individual self-reported health status					Individual self-reported health status				
Good/Very Good	Ref				Good/Very Good	Ref			
Moderate	0.721	0.001**	0.597	0.870	Moderate	0.717	0.001**	0.588	0.874
Bad/Very Bad	0.602	0.001**	0.441	0.821	Bad/Very Bad	0.576	0.002**	0.405	0.820
Age at menarche in years					Age at menarche in years				
Early (<12)	Ref				Early (<12)	Ref			
Normal (12–15)	1.615	0.037*	1.030	2.530	Normal (12–15)	1.755	0.023*	1.080	2.850
Late (>15)	1.341	0.228	0.832	2.159	Late (>15)	1.548	0.099	0.921	2.602
Household mass media exposure					Household mass media exposure				
No exposure	Ref				No exposure	Ref			
Less than once a week	1.279	0.051	0.998	1.638	Less than once a week	1.403	0.010*	1.086	1.812
At least once a week	1.304	0.029*	1.028	1.654	At least once a week	1.342	0.021*	1.045	1.722
Household WASH status					Household WASH status				
Unimproved WASH	Ref				Unimproved WASH	Ref			
Improved WASH	1.131	0.248	0.918	1.393	Improved WASH	1.185	0.091	0.973	1.442
Household wealth status					Household wealth status				
Poorest	Ref				Poorest	Ref			
Poorer	1.297	0.009**	1.068	1.576	Poorer	1.289	0.010*	1.062	1.563
Middle	1.420	0.011*	1.085	1.857	Middle	1.500	0.003*	1.145	1.964
Richer	2.213	<0.001***	1.573	3.114	Richer	2.354	<0.001***	1.655	3.347
Richest	5.269	<0.001***	3.276	8.475	Richest	5.867	<0.001***	3.616	9.519
Individual educational level					Individual educational level				
No education	Ref				No education	Ref			
Primary	1.368	0.003**	1.110	1.686	Primary	1.385	0.003*	1.114	1.722
Secondary	2.004	<0.001***	1.609	2.494	Secondary	2.027	<0.001***	1.597	2.572
Higher	5.917	<0.001***	2.665	13.135	Higher	6.639	<0.001***	3.077	14.323
Place of residence					Place of residence				
urban	Ref				Urban	Ref			
rural	0.698	0.017*	0.519	0.938	rural	0.735	0.023*	0.563	0.958
Region					Region				
Western	Ref				Western	Ref			
Central	1.576	0.039*	1.022	2.431	Central	1.736	0.020*	1.089	2.766
Greater accra	1.644	0.057	0.985	2.746	Greater accra	1.904	0.019*	1.114	3.253
Volta	1.207	0.424	0.761	1.914	Volta	1.152	0.561	0.714	1.857
Eastern	1.761	0.027*	1.068	2.904	Eastern	1.917	0.015*	1.132	3.248
Ashanti	1.667	0.051	0.998	2.783	Ashanti	1.894	0.021*	1.101	3.255
Western north	3.008	<0.001***	1.913	4.729	Western north	3.215	<0.001****	1.979	5.223
Ahafo	3.714	<0.001***	2.044	6.749	Ahafo	4.089	<0.001***	2.242	7.460
Bono	6.109	<0.001***	3.472	10.748	Bono	6.650	<0.001***	3.584	12.337
Bono east	2.484	<0.001***	1.556	3.965	Bono east	2.865	<0.001***	1.746	4.699
Oti	1.081	0.720	0.706	1.653	Oti	1.062	0.798	0.670	1.683
Northern	0.915	0.706	0.575	1.454	Northern	0.934	0.786	0.570	1.529
Savannah	2.324	0.004**	1.311	4.119	Savannah	2.470	0.001*	1.415	4.312
Northeast	2.643	0.005**	1.336	5.227	Northeast	3.807	<0.001***	1.902	7.619
Upper east	4.152	<0.001***	2.463	6.998	Upper east	4.473	<0.001***	2.561	7.814
Upper west	4.319	<0.001***	2.493	7.481	Upper west	4.667	<0.001***	2.617	8.322
Individual age in years					Individual age in years				
15–19	Ref				15–19	Ref			
20–24	1.641	0.003**	1.182	2.277	20–24	1.686	0.002**	1.205	2.359
25–29	1.103	0.579	0.780	1.559	25–29	1.082	0.657	0.763	1.535
30–34	0.740	0.090	0.523	1.048	30–34	0.706	0.058	0.492	1.012
35–39	0.625	0.014*	0.430	0.908	35–39	0.584	0.006**	0.399	0.854
40–44	0.488	<0.001***	0.341	0.699	40–44	0.443	<0.001***	0.307	0.639
45–49	0.379	<0.001***	0.257	0.558	45–49	0.333	<0.001***	0.225	0.492
/cut1	−2.924		−3.562	−2.286	/cut1	−2.961		−3.620	−2.301
/cut2	−0.647		−1.298	0.004	/cut2	−0.586		−1.258	0.086
					PSU var(_cons)	0.505		0.381	0.670
AOR = Adjusted Odds Ratio, ****p < 0.0001, ***p < 0.001, **p < 0.01, *p < 0.05, ref = reference category, WASH = Water, Sanitation and Hygiene, ll(model) = −4,093.0287, ll(null) = −5,084.7445, McFadden R2 = 0.1950, Pseudo R2 = 0.1950, AIC = 8,272.057, BIC = 8,589.23					AOR = Adjusted Odds Ratio, ****p < 0.0001, ***p < 0.001, **p < 0.01, *p < 0.05, ref = reference category, WASH = Water, Sanitation and Hygiene, ll(model) = −4,000.998, AIC = 8,089.997, BIC = 8,414.546, Inter cluster correlation (ICC) = 0.1331435, Variance Component (*τ*²) = 0.5053021, Standard Error (SE) of Variance = 0.0730378, Median Odds Ratio (MOR) = 1.97, McFadden’s R² = 0.21313686				

Furthermore, women living in communities with higher average education were more likely to use higher-category menstrual products (AOR = 1.76, 95% CI: 1.21–2.56, *p* = 0.003). Also, household exposure to mass media at least once weekly increased the odds of higher product use (AOR = 1.34, 95% CI: 1.05–1.72, *p* = 0.021); less-than-weekly exposure also increased odds but was smaller (AOR = 1.40, 95% CI: 1.09–1.81, *p* = 0.010). There was a strong, monotonic wealth gradient. Compared with the poorest quintile, odds of higher product use were greater in progressively wealthier households: poorer AOR = 1.29 (95% CI: 1.06–1.56, *p* = 0.010), middle AOR = 1.50 (95% CI: 1.15–1.96, *p* = 0.003), richer AOR = 2.35 (95% CI: 1.66–3.35, *p* < 0.001), and richest AOR = 5.87 (95% CI: 3.62–9.52, *p* < 0.001) (Table 2). Education was strongly associated with higher product use: primary AOR = 1.39 (95% CI: 1.11–1.72, *p* = 0.003), secondary AOR = 2.03 (95% CI: 1.60–2.57, *p* < 0.001), and higher education AOR = 6.64 (95% CI: 3.08–14.32, *p* < 0.001). Relative to adolescents aged 15–19, young adults 20–24 had increased odds of higher product use (AOR = 1.69, 95% CI: 1.21–2.36, *p* = 0.002), while older age groups had progressively lower odds (e.g., 35–39: AOR = 0.58, 95% CI: 0.40–0.85, *p* = 0.006; 40–44: AOR = 0.44, 95% CI: 0.31–0.64, *p* < 0.001; 45–49: AOR = 0.33, 95% CI: 0.23–0.49, *p* < 0.001).

Moreover, compared with never-in-union, currently partnered women had lower odds of higher product use (AOR = 0.58, 95% CI: 0.43–0.77, *p* < 0.001); widowed/divorced/separated women also had reduced odds (AOR = 0.58, 95% CI: 0.40–0.86, *p* = 0.006). Also, women reporting moderate health had lower odds than those reporting good/very good health (AOR = 0.72, 95% CI: 0.59–0.87, *p* = 0.001); those reporting bad/very bad health were even less likely to use higher-category products (AOR = 0.58, 95% CI: 0.41–0.82, *p* = 0.002). However, menarche in the normal age range (12–15 years) was associated with higher odds vs. early menarche (AOR = 1.76, 95% CI: 1.08–2.85, *p* = 0.023). Nonetheless, rural residence was associated with lower odds of higher product use compared with urban residence (AOR = 0.74, 95% CI: 0.56–0.96, *p* = 0.023). Also, marked regional heterogeneity persisted after adjustment. For example, relative to Western region, women in Bono had markedly higher odds (AOR = 6.65, 95% CI: 3.58–12.34, *p* < 0.001), Upper East (AOR = 4.47, 95% CI: 2.56–7.81, *p* < 0.001), and Western North (AOR = 3.22, 95% CI: 1.98–5.22, *p* < 0.001) (Table 2).

### Predictive ability of single-level and multi-level ordered logistic regression models to predict menstrual product use

#### Somers' D and concordance (C) index with ordered menstrual product use variable

Table 3 presents Somers' D and the C-index values for both the single-level and multilevel ordered logistic regression models predicting menstrual product use. The single-level model achieved a Somers' D of 0.648 (95% CI: 0.626–0.669, *p* < 0.001), corresponding to a C-index of 0.824. The multilevel random-intercept model produced nearly identical results, with a Somers' D of 0.648 (95% CI: 0.627–0.670, *p* < 0.001) and a C-index of 0.824. These findings indicate that both models possess excellent discriminative ability, with over 82% concordance between predicted and observed rankings of menstrual product use. The minimal difference in performance suggests that while the multilevel model provides a better statistical fit and accounts for clustering (as reported in Table 2), it does not materially alter predictive accuracy compared with the single-level specification (Table 3).

**Table 3:** Somers’ D and C-index describing the predictive probability of the single and multi-level ordered models.

Single level ordered logistic regression (Left panel)				Multi-level ordered logistic regression (Right panel)			
Coefficient	*p*-value	95% CI		Coefficient	*p*-value	95% CI	
0.6475524	<0.001***	0.6260302	0.6690747	0.6480865	<0.001***	0.6266251	0.669548
C-index: 0.8237762				C-index: 0.82404325			
***p < 0.001				***p < 0.001			

#### The distribution of predicted values by ordered menstrual product use variable

**Figure 1:**
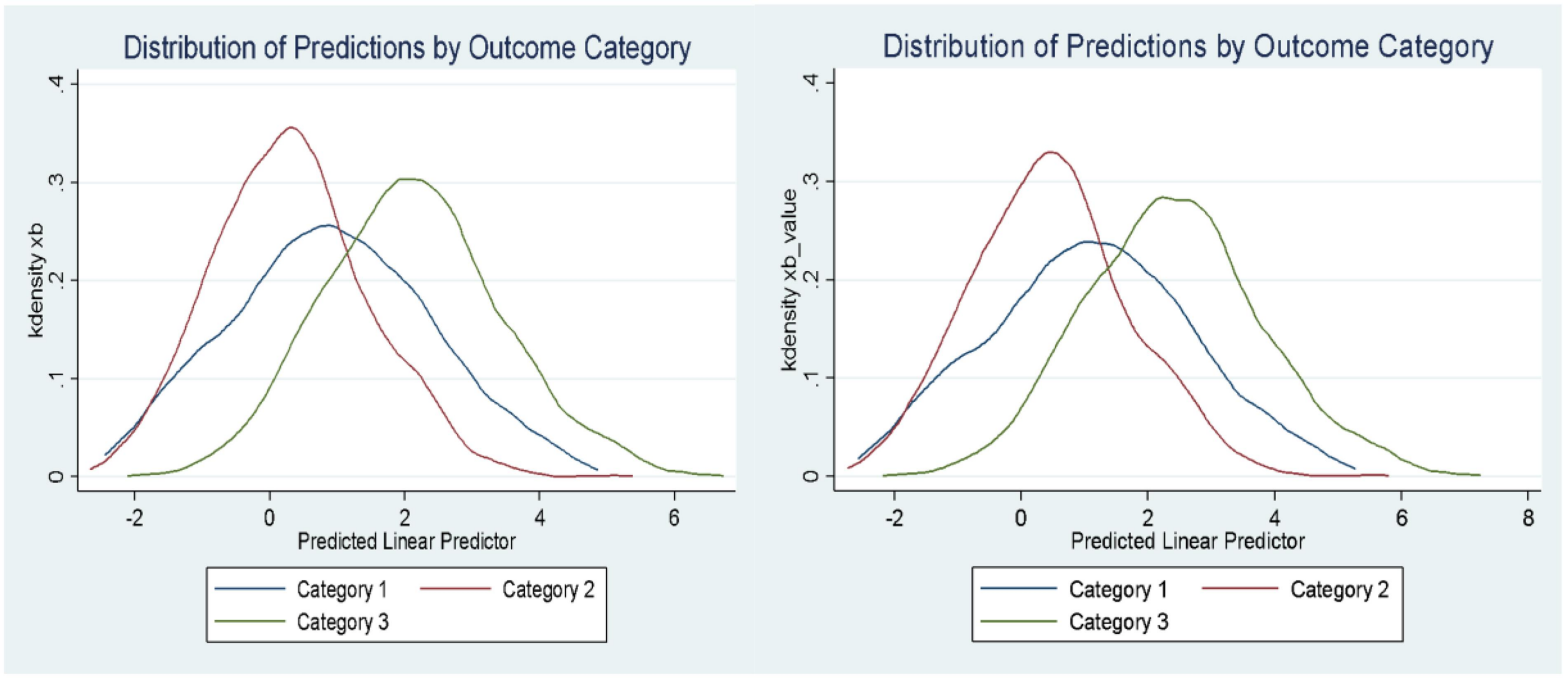
Kernel density nplot of predicted values across outcome estimated from **(A)** single-level ordered logistic regression (left panel), **(B)** multi-level ordered logistic regression (right panel). Category 1 = Nothing/Other, Category 2 = Traditional, Category 3 = Modern.

Figure 1 shows the distribution of predicted linear predictors from the single-level (panel A) and multi-level (panel B) ordered logistic regression models. In both models, the density curves demonstrate clear separation across the three categories of menstrual product use. Women using nothing/other (Category 1) had predicted values concentrated at the lower end of the scale, while those using traditional products (Category 2) were distributed around the mid-range. By contrast, the modern product users (Category 3) were shifted towards higher predicted values, with a distinctly rightward distribution. The overlapping yet distinct peaks across categories indicate that the models adequately differentiate between non-use, traditional use, and modern use. The similarity between the single-level and multi-level plots suggests that accounting for clustering at the primary sampling unit did not materially alter the predicted probability distribution. However, the multilevel model (panel B) displayed slightly sharper separation between traditional and modern product users, consistent with the improved model fit statistics reported earlier (Figure 1).

### Single-level and multi-level logistic regression model of determinants of modern menstrual product use (traditional/others/nothing vs. modern)

Table 4 presents the results of the single-level and multi-level logistic regression models examining factors associated with modern menstrual product use compared to traditional or no use. At the community level, higher education within communities significantly increased the likelihood of modern product use in both the single-level model (AOR = 1.61, 95% CI: 1.07–2.42, *p* = 0.022) and the multi-level model (AOR = 1.79, 95% CI: 1.21–2.66, *p* = 0.004). Similarly, greater household mass media exposure was positively associated with modern product use, particularly for those exposed at least once a week (single-level: AOR = 1.36, 95% CI: 1.06–1.75, *p* = 0.017; multi-level: AOR = 1.43, 95% CI: 1.09–1.88, *p* = 0.009). At the individual level, marital status showed strong associations: compared to women never in union, those married, or cohabiting, had significantly lower odds of using modern products (multi-level: AOR = 0.60, 95% CI: 0.45–0.79, *p* < 0.001). Similarly, widowed, divorced, or separated women had reduced odds (AOR = 0.60, 95% CI: 0.40–0.88, *p* = 0.009). Health status was also influential, with women reporting poor health less likely to use modern products (AOR = 0.54, 95% CI: 0.38–0.78, *p* = 0.001). Reproductive history also mattered. Compared to those with early menarche (<12 years), women with menarche between ages 12–15 had higher odds of modern use (multi-level AOR = 1.81, 95% CI: 1.11–2.95, *p* = 0.017).

**Table 4:** Single-level and multi-level logistic regression model of determinants of modern menstrual product use.

Single-level logistic regression model of determinants of modern menstrual product use (Left panel)					Multi-level logistic regression model of determinants of modern menstrual product use (Right panel)				
Variables	AOR	*P*-value	95% CI		Variables	AOR	*P*-value	95% CI	
Community mass media exposure	1.221	0.272	0.855	1.744	Community mass media exposure	1.214	0.337	0.817	1.802
Community education	1.613	0.022*	1.073	2.424	Community education	1.793	0.004*	1.209	2.658
Community poverty	0.855	0.175	0.682	1.072	Community poverty	0.837	0.115	0.670	1.044
Individual marital status					Individual marital status				
Never in Union	Ref				Never in Union	Ref			
Married/Living with Partner	0.620	0.001**	0.467	0.823	Married/Living with Partner	0.597	<0.001***	0.450	0.792
Widowed/Divorced/Separated	0.625	0.016*	0.426	0.916	Widowed/Divorced/Separated	0.597	0.009**	0.404	0.879
Individual self-reported health status					Individual self-reported health status				
Good/Very Good	Ref				Good/Very Good	Ref			
Moderate	0.707	0.001**	0.582	0.859	Moderate	0.704	0.001**	0.572	0.867
Bad/Very Bad	0.566	0.001**	0.408	0.785	Bad/Very Bad	0.543	0.001**	0.379	0.778
Age at menarche in years					Age at menarche in years				
Early (<12)	Ref				Early (<12)	Ref			
Normal (12–15)	1.682	0.028*	1.058	2.675	Normal (12–15)	1.813	0.017*	1.112	2.953
Late (>15)	1.419	0.164	0.867	2.321	Late (>15)	1.654	0.061	0.977	2.799
Household mass media exposure					Household mass media exposure				
No exposure	Ref				No exposure	Ref			
Less than once a week	1.314	0.047*	1.004	1.719	Less than once a week	1.473	0.007**	1.113	1.948
At least once a week	1.360	0.017*	1.056	1.751	At least once a week	1.432	0.009**	1.092	1.877
Household WASH status					Household WASH status				
Unimproved WASH	Ref				Unimproved WASH	Ref			
Improved WASH	1.143	0.220	0.923	1.415	Improved WASH	1.202	0.070	0.985	1.466
Household wealth status					Household wealth status				
Poorest	Ref				Poorest	Ref			
Poorer	1.342	0.008**	1.081	1.666	Poorer	1.346	0.008**	1.080	1.678
Middle	1.479	0.007**	1.114	1.963	Middle	1.576	0.002**	1.177	2.110
Richer	2.301	<0.001***	1.622	3.262	Richer	2.459	<0.001***	1.708	3.539
Richest	5.385	<0.001***	3.331	8.703	Richest	6.082	<0.001***	3.716	9.951
Individual educational level					Individual educational level				
No education	Ref				No education	Ref			
Primary	1.418	0.002**	1.141	1.762	Primary	1.462	0.001**	1.162	1.839
Secondary	2.070	<0.001***	1.666	2.571	Secondary	2.153	<0.001***	1.698	2.729
Higher	6.063	<0.001***	2.719	13.518	Higher	6.925	<0.001***	3.156	15.191
Place of residence					Place of residence				
Urban	Ref				Urban	Ref			
Rural	0.696	0.019*	0.514	0.941	Rural	0.726	0.022*	0.552	0.955
Region					Region				
Western	Ref				Western	Ref			
Central	1.576	0.047*	1.007	2.468	Central	1.747	0.024*	1.075	2.839
Greater accra	1.709	0.045*	1.012	2.888	Greater accra	2.023	0.012*	1.166	3.510
Volta	1.190	0.484	0.731	1.936	Volta	1.119	0.664	0.675	1.855
Eastern	1.786	0.028*	1.063	3.001	Eastern	1.969	0.016*	1.136	3.413
Ashanti	1.771	0.032*	1.050	2.984	Ashanti	2.050	0.012*	1.174	3.578
Western north	3.211	<0.001***	2.011	5.127	Western north	3.506	<0.001***	2.110	5.824
Ahafo	4.031	<0.001***	2.182	7.447	Ahafo	4.550	<0.001***	2.439	8.487
Bono	6.439	<0.001***	3.618	11.461	Bono	7.173	<0.001***	3.790	13.578
Bono east	2.629	<0.001***	1.604	4.310	Bono east	3.074	<0.001***	1.822	5.188
Oti	1.051	0.829	0.669	1.651	Oti	1.027	0.915	0.627	1.684
Northern	0.843	0.500	0.513	1.385	Northern	0.855	0.564	0.503	1.453
Savannah	2.485	0.003**	1.363	4.531	Savannah	2.654	0.001**	1.468	4.797
Northeast	2.736	0.008**	1.307	5.729	Northeast	4.025	<0.001***	1.882	8.608
Upper east	4.535	<0.001***	2.639	7.792	Upper east	4.995	<0.001***	2.782	8.968
Upper west	4.963	<0.001***	2.816	8.746	Upper west	5.469	<0.001***	2.983	10.028
Individual age in years					Individual age in years				
15–19	Ref				15–19	Ref			
20–24	1.654	0.004**	1.180	2.318	20–24	1.705	0.003**	1.201	2.421
25–29	1.080	0.668	0.759	1.537	25–29	1.045	0.811	0.731	1.491
30–34	0.699	0.044*	0.492	0.991	30–34	0.648	0.019*	0.450	0.932
35–39	0.585	0.005**	0.401	0.853	35–39	0.529	0.001**	0.361	0.776
40–44	0.449	<0.001***	0.310	0.649	40–44	0.400	<0.001***	0.274	0.583
45–49	0.341	<0.001***	0.228	0.508	45–49	0.287	<0.001***	0.190	0.433
					PSU var(_cons)	0.578		0.442	0.755
AOR = Adjusted Odds Ratio, ***p < 0.001, **p < 0.01, *p < 0.05, ref = reference category, WASH = Water, Sanitation and Hygiene, ll(null) = −4,459.489, ll(model) = −3,422.413, McFadden R2 = 0.233, Pseudo R2 = 0.2326, AIC = 6,928.826 BIC = 7,238.622.					AOR = Adjusted Odds Ratio, ***p < 0.001, **p < 0.01, *p < 0.05, ref = reference category, WASH = Water, Sanitation and Hygiene, ll(model) = −3,319.095, AIC = 6,724.19, BIC = 7,041.362, Inter cluster correlation (ICC) = 0.1495004, Variance Component (*τ*²) = 0.5783, Standard Error (SE) of Variance = 0.0790, Median Odds Ratio (MOR) = 1.89, McFadden’s R² = 0.19124736				

Household wealth and education demonstrated strong dose–response relationships. The richest women had six-fold greater odds of using modern products compared with the poorest (multi-level AOR = 6.08, 95% CI: 3.72–9.95, *p* < 0.001). Similarly, women with higher education were substantially more likely to use modern products (multi-level AOR = 6.93, 95% CI: 3.16–15.19, *p* < 0.001). Place of residence was also significant: rural women had reduced odds of modern product use compared to urban women (multi-level AOR = 0.73, 95% CI: 0.55–0.96, *p* = 0.022). Regional disparities were pronounced, with women in Bono (AOR = 7.17, 95% CI: 3.79–13.58, *p* < 0.001) and Upper West (AOR = 5.47, 95% CI: 2.98–10.03, *p* < 0.001) being far more likely to use modern products than those in the Western region. Finally, age effects showed a gradient: compared to adolescents (15–19 years), older women had consistently lower odds of modern product use. For example, women aged 40–44 had AOR = 0.40 (95% CI: 0.27–0.58, *p* < 0.001), while those aged 45–49 had the lowest odds (AOR = 0.29, 95% CI: 0.19–0.43, *p* < 0.001). The model fit statistics indicated strong predictive performance. The multi-level model showed better fit (AIC = 6,724.19; BIC = 7,041.36) compared with the single-level model (AIC = 6,928.83; BIC = 7,238.62). The intraclass correlation (ICC = 0.15) confirmed that 15% of the variance in modern menstrual product use was attributable to between-cluster differences. The variance component (*τ*² = 0.58, SE = 0.079) and median odds ratio (MOR = 1.89) further highlighted the influence of contextual factors (Table 4).

### Predictive ability of single-level and multi-level logistic regression models to predict modern menstrual product use

**Figure 2:**
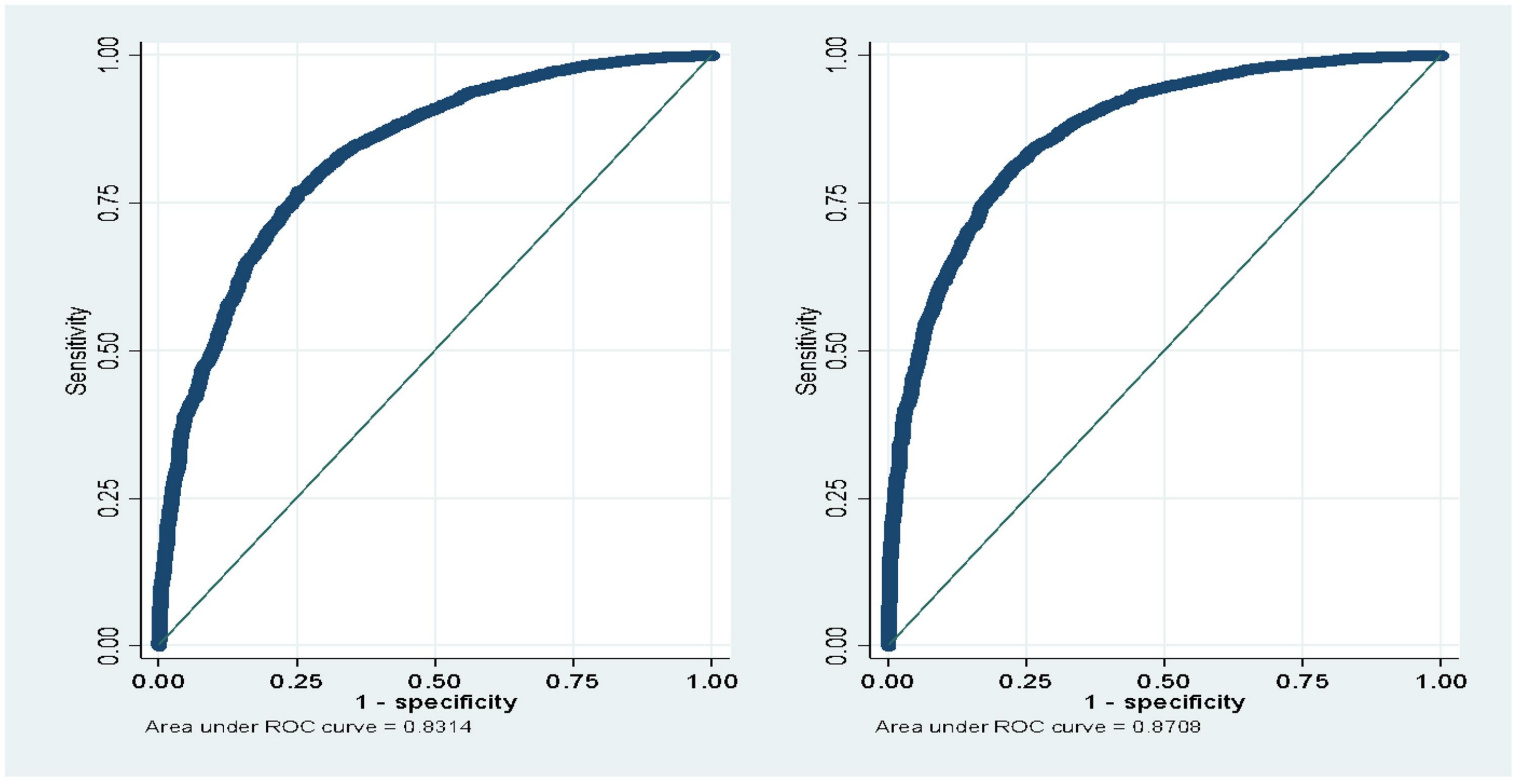
Predictive probabilities of **(A)** single-level model (left panel) and **(B)** multi-level model (right panel) to determine the predictors of modern menstrual product use.

The discriminative performance of the models was assessed using receiver operating characteristic (ROC) curves (Figure 2). The single-level logistic regression model demonstrated good predictive ability with an area under the ROC curve (AUC) of 0.83. The multi-level logistic regression model achieved a higher AUC of 0.87, indicating superior predictive accuracy compared to the single-level specification. This improvement underscores the contribution of cluster-level factors, as captured by the random intercept in the multi-level model, in explaining variation in modern menstrual product use. The findings align with the significant intraclass correlation (ICC = 0.15), suggesting that contextual and household-level influences meaningfully improve predictive power beyond individual-level determinants (Figure 2).

### Non-linear multivariate logistic decomposition model to predict modern menstrual product use by residence

The decomposition analysis by residence in Table 5a below, revealed that differences in characteristics accounted for 63% of the rural–urban inequality in modern menstrual product use, while differences in coefficients explained 37%. Among the endowments, household wealth status contributed the largest share. Women from wealthier households, particularly those in the richest quintile, had significantly higher use of modern menstrual products compared to their rural counterparts (Coef. = 0.040, *p* < 0.001; 32.9%). Education also mattered, with secondary and higher education positively contributing to the gap, while primary education reduced it. Regional variation was notable; for instance, women in Greater Accra and Ashanti explained a sizable part of the residence gap, whereas women in Upper East and Upper West reduced it (Table 5b). Turning to coefficients, the unexplained component showed that marital status played an important role. Married women in rural areas exhibited lower returns to characteristics compared to their urban peers (Coef. = −0.033, *p* < 0.05; −27.3%). Similarly, returns to wealth status differed; poorer women had a significantly reduced likelihood of use compared to their urban counterparts. Age also contributed meaningfully; rural women aged 20–29 had stronger positive coefficients, suggesting higher predicted use than their urban peers of similar age (Table 5c).

**Table 5a:** Decomposition analysis by residence in the predictors of modern menstrual product use (total difference).

Modern menstrual product use	Coef.	*p*-value	95% CI	Pct.
Due to Endowments (E)	0.07658	<0.001	(0.03760–0.11556)	62.96
Due to Coefficients (C)	0.04506	0.047	(0.00056–0.08955)	37.04
Total (R)	0.12164	<0.001	(0.10340–0.13988)	100.00

Reference group (A): Residence (rural) = 1, Mean = 0.9294.

Comparison group (B): Residence (urban) = 0, Mean = 0.8079.

**Table 5b:** Decomposition analysis by residence [differences due to characteristics (E)].

Variables	Coef.	*p*-value	95% CI	Pct.
Community mass media exposure	0.00420	0.668	−0.01498–0.02338	3.45
Community education	0.00275	0.787	−0.01715–0.02265	2.26
Community poverty	−0.01068	0.641	−0.05558–0.03423	−8.78
Marital status (ref = never married)				
Married/Living with Partner	0.00641	0.001	0.00273–0.01009	5.27
Widowed/Divorced/Separated	−0.00042	0.011	−0.00074–−0.00009	−0.34
Health status (ref = good)				
Moderate	−0.00004	0.028	−0.00007–−0.00000	−0.03
Bad/Very Bad	0.00104	0.060	−0.00004–0.00213	0.86
Age at menarche [ref = early (<12)]				
Normal (12–15)	0.00175	0.101	−0.00034–0.00384	1.44
Late (>15)	−0.00154	0.424	−0.00532–0.00224	−1.27
Mass media exposure (ref = no exposure)				
Less than once/week	−0.00150	0.125	−0.00342–0.00042	−1.24
At least once/week	0.00308	0.372	−0.00369–0.00984	2.53
WASH status (ref = unimproved WASH)				
Improved WASH	0.00010	0.985	−0.01057–0.01077	0.08
Wealth status (ref = poorest)				
Poorer	0.00232	0.585	−0.00601–0.01065	1.91
Middle	0.00044	0.387	−0.00056–0.00144	0.36
Richer	0.01231	0.007	0.00331–0.02132	10.12
Richest	0.04002	<0.001	0.02326–0.05678	32.90
Education level (ref = no education)				
Primary	−0.00246	0.031	−0.00470–−0.00023	−2.03
Secondary	0.00591	<0.001	0.00308–0.00873	4.86
Higher	0.01365	<0.001	0.00597–0.02134	11.22
Region (ref = Western)				
Central	−0.00019	0.003	−0.00031–−0.00007	−0.16
Greater Accra	0.01655	0.001	0.00660–0.02651	13.61
Volta	−0.00090	0.152	−0.00213–0.00033	−0.74
Eastern	−0.00184	0.004	−0.00309–−0.00058	−1.51
Ashanti	0.00301	0.001	0.00124–0.00478	2.47
Western North	−0.00377	<0.001	−0.00583–−0.00172	−3.10
Ahafo	−0.00125	<0.001	−0.00194–−0.00056	−1.03
Bono	0.00174	<0.001	0.00096–0.00252	1.43
Bono East	0.00004	<0.001	0.00002–0.00006	0.03
Oti	−0.00092	0.169	−0.00222–0.00039	−0.75
Northern	−0.00012	0.807	−0.00104–0.00081	−0.09
Savannah	−0.00118	0.151	−0.00278–0.00043	−0.97
North East	−0.00338	<0.001	−0.00492–−0.00185	−2.78
Upper East	−0.00741	0.001	−0.01196–−0.00285	−6.09
Upper West	−0.00385	0.002	−0.00633–−0.00136	−3.16
Age group (ref = 15–19)				
20–24	0.00110	0.001	0.00044–0.00176	0.91
25–29	0.00084	0.058	−0.00003–0.00171	0.69
30–34	−0.00000	0.983	−0.00013–0.00012	0.00
35–39	−0.00009	0.732	−0.00063–0.00044	−0.08
40–44	0.00030	0.213	−0.00017–0.00078	0.25
45–49	0.00053	0.036	0.00003–0.00102	0.43

Coef., beta coefficient from logit estimates; R, Endowment/Characteristics; E, Differences due to Characteristics; Pct., percentage contribution; Ref, reference category.

**Table 5c:** Decomposition analysis by residence [differences due to coefficients (C)].

Variables	Coef.	*p*-value	95% CI	Pct.
Community mass media exposure	−0.00007	0.981	−0.00594–0.00580	−0.06
Community education	0.00560	0.136	−0.00177–0.01296	4.60
Community poverty	−0.00169	0.877	−0.02312–0.01973	−1.39
Marital status (ref = never married)				
Married/Living with Partner	−0.03325	0.047	−0.06613–−0.00037	−27.34
Widowed/Divorced/Separated	−0.00545	0.166	−0.01316–0.00226	−4.48
Health status (ref = good)				
Moderate	0.00021	0.957	−0.00768–0.00811	0.18
Bad/Very Bad	−0.00048	0.767	−0.00362–0.00267	−0.39
Age at menarche [ref = early(<12)]				
Normal (12–15)	0.01662	0.569	−0.04054–0.07377	13.66
Late (>15)	0.00049	0.972	−0.02656–0.02753	0.40
Mass media exposure (ref = no exposure)				
Less than once/week	0.00435	0.417	−0.00615–0.01484	3.57
At least once/week	−0.00787	0.671	−0.04421–0.02847	−6.47
WASH status (ref = unimproved WASH)				
Improved WASH	−0.01203	0.211	−0.03088–0.00682	−9.89
Wealth status (ref = poorest)				
Poorer	−0.01335	0.128	−0.03057–0.00386	−10.98
Middle	−0.00058	0.924	−0.01262–0.01145	−0.48
Richer	0.00400	0.375	−0.00483–0.01283	3.29
Richest	0.00467	0.154	−0.00174–0.01108	3.84
Education level (ref = no education)				
Primary	0.00317	0.431	−0.00473–0.01108	2.61
Secondary	0.00415	0.736	−0.02002–0.02833	3.41
Higher	−0.00401	0.296	−0.01153–0.00351	−3.30
Region (ref = Western)				
Central	0.00793	0.116	−0.00195–0.01780	6.52
Greater Accra	0.00161	0.209	−0.00090–0.00412	1.32
Volta	0.00279	0.335	−0.00288–0.00846	2.29
Eastern	0.00880	0.070	−0.00072–0.01831	7.23
Ashanti	0.02080	0.023*	0.00285–0.03874	17.10
Western North	0.00284	0.201	−0.00151–0.00719	2.33
Ahafo	0.00031	0.846	−0.00283–0.00345	0.26
Bono	0.00222	0.226	−0.00137–0.00580	1.82
Bono East	0.00436	0.037*	0.00026–0.00845	3.58
Oti	0.00249	0.149	−0.00090–0.00589	2.05
Northern	0.00212	0.607	−0.00595–0.01020	1.74
Savannah	−0.00067	0.732	−0.00448–0.00315	−0.55
North East	0.00508	0.002	0.00182–0.00835	4.18
Upper East	0.00382	0.342	−0.00405–0.01168	3.14
Upper West	−0.00028	0.912	−0.00525–0.00468	−0.23
Age group (ref = 15–19)				
20–24	0.01634	0.012	0.00365–0.02903	13.43
25–29	0.01267	0.017	0.00222–0.02312	10.42
30–34	0.00862	0.116	−0.00214–0.01939	7.09
35–39	0.00943	0.085	−0.00132–0.02018	7.76
40–44	0.00838	0.092	−0.00136–0.01812	6.89
45–49	0.00440	0.202	−0.00237–0.01116	3.62

Coef., beta coefficient from logit estimates, *p < 0.05, R, Endowment/Characteristics; C, Coefficients/Returns; Pct., percentage contribution; Ref, reference category.

For clarity, the largest single contributors to the rural–urban gap on the endowment side were household wealth (richest quintile, 32.9%), residence in Greater Accra (13.6%), and higher education (11.2%), whereas on the coefficient side the differential returns among women in Ashanti (17.1%) and the lower returns to marriage among rural women (−27.3%) were most prominent.

## Discussion

### Summary of findings

The study demonstrated that use of modern menstrual products among reproductive-aged women is strongly associated with socioeconomic, demographic, and contextual factors. Uptake was highest among younger women, those with favourable health status, higher education, and greater household wealth, with clear gradients observed across education and wealth quintiles. Urban residence, community-level education, regular media exposure, and improved WASH access further enhanced the likelihood of use, while rural women, those in poorer households, and women with limited education reported lower uptake. Multilevel modelling confirmed substantial cluster-level variation, with 13%–15% of differences attributable to community effects, underscoring the influence of contextual environments. Regional disparities persisted, with Greater Accra and Ashanti showing higher prevalence, while northern and recently created regions lagged. Decomposition analyses revealed that inequalities in menstrual product use were primarily driven by compositional differences in wealth and education, though differential returns to age, marital status, and WASH access also contributed.

### Proportion of modern menstrual product use

The finding that women from households with improved water, sanitation and hygiene (WASH) facilities reported significantly higher use of modern menstrual products underscores the critical role of infrastructure in enabling safe menstrual health practices, echoing evidence from Ghana and elsewhere in sub-Saharan Africa where inadequate WASH is linked to poor uptake (11–14). Wealth-related inequalities were evident, with women in the richest households reporting more than double the use compared to the poorest, reflecting affordability challenges like those reported by Anaba et al. (5) in Ghana and Phillips-Howard et al. (15) in Kenya. Educational attainment also showed a strong gradient, with secondary education associated with the highest uptake, aligning with Appiah-Agyekum et al. (16) and comparable findings from Ethiopia, where higher education improved menstrual hygiene practices (17). Urban residence and regional disparities further reinforced structural inequities, with Greater Accra and Ashanti showing higher uptake relative to northern and newly created regions, consistent with national surveys (18). Finally, the age distribution revealed that adolescents were the most likely to use modern products, while older women relied more on reusable materials, reflecting patterns observed in other Ghanaian studies (5, 19).

### Determinants of menstrual product use

This study's findings emphasise that individual determinants, particularly wealth and education, remain the most potent predictors of modern menstrual product use in Ghana. Women from the richest households were over six times more likely to adopt modern menstrual products compared to their poorest counterparts, while those with higher education had nearly seven-fold greater odds. These associations highlight the dual roles of economic capacity and cognitive empowerment in shaping menstrual health behaviours. Wealth is likely associated with improved affordability and consistent access to sanitary pads or menstrual cups, while education may equip women with knowledge and skills that support informed health decisions, help challenge stigma, and facilitate hygienic practices. These patterns are consistent with multi-country evidence showing that socioeconomic advantage is substantially associated with menstrual product use across low- and middle-income contexts (20, 21). The results also affirm the applicability of the “health capability framework,” where material resources and knowledge together determine the extent to which women can translate opportunities into positive health practices. Additionally, the strong, dose-response relationship between education and modern product use is consistent with the broader evidence base on health education. A randomized trial in Zimbabwe demonstrated that well-designed educational interventions can effectively improve reproductive health knowledge and behaviour among adolescents (22). This underscores the potential of school-based programs to drive sustained improvements in menstrual health practices.

Marital status and reported health status also emerged as important individual-level predictors. Married, cohabiting, or formerly married women had significantly lower odds of modern product use than those never in union. This may reflect competing household responsibilities, economic dependency on partners, and entrenched cultural norms that relegate women's health needs to a lower priority. In patriarchal settings, marital arrangements may restrict women's autonomy in making decisions about personal health expenditures, including menstrual products. These findings mirror those from Tanzania and India, where spousal dynamics, cultural expectations, and marital roles were found to limit menstrual hygiene choices (23, 24). Additionally, women reporting poor self-rated health were significantly less likely to use modern products, suggesting that health vulnerabilities constrain agency in prioritising menstrual care. Such women may be more preoccupied with managing chronic illness or other pressing health needs, with menstruation receiving less attention.

Reproductive history also shaped menstrual management. Women with menarche between ages 12–15 were significantly more likely to use modern products compared with those with early menarche. Early menarche often coincides with limited maturity and preparedness, potentially leading to reliance on improvised absorbents and perpetuating long-term patterns of unhygienic practices. Conversely, girls experiencing menarche later may be more exposed to school-based education and supportive information, fostering readiness for modern product use. Age also demonstrated a strong gradient, with younger women (15–19 years) more likely to adopt modern products, while older women, particularly those aged 40–49, showed sharply reduced odds. This may reflect generational differences in awareness, cultural acceptance, and exposure to menstrual health campaigns, as younger cohorts are increasingly targeted by education and advocacy initiatives. Studies confirm that older women are less likely to transition to modern products, relying instead on traditional materials (5, 25). Nonetheless, the higher prevalence of modern product use among younger women may reflect their greater exposure to school-based health education and peer networks. This is particularly important given that, as documented in Kenya, menstruation-related stigma can severely restrict girls' educational participation and spatial mobility (26). Interventions targeting this demographic may therefore have a multiplier effect on their educational and social outcome.

Community-level factors played a substantial role. Women residing in communities with higher education attainment were nearly twice as likely to use modern products, underscoring the influence of collective social capital and shared knowledge environments. Similarly, frequent exposure to mass media, especially weekly engagement, significantly increased odds of adoption, indicating that information diffusion through radio, television, and community campaigns normalises menstrual health practices and reduces stigma. These findings align with studies in West Africa that highlighted mass media as a crucial vehicle for improving knowledge and shifting attitudes on menstruation (27–29). Also, geographic and regional disparities were pronounced. Rural women had substantially lower odds of modern product use than urban women, reflecting barriers such as limited market availability, infrastructural challenges, and sociocultural norms that stigmatise menstruation. Regional analysis further revealed striking contrasts, with Bono and Upper West regions demonstrating substantially higher odds compared to the Western region, we hypothesise that this may reflect uneven distribution of programmes, policy focus, or differing cultural receptivity, although the cross-sectional design cannot test these explanations directly.

### Decomposition by place of residence

Decomposition analysis confirmed that differences in household wealth and education accounted for more than half of the rural–urban gap, while coefficients highlighted structural disadvantages whereby rural women derived fewer returns from similar characteristics. For instance, rural married women with some education were less likely to convert these attributes into modern product use compared with their urban peers. This mirrors wider findings across sub-Saharan Africa that rural disadvantage extends beyond material deprivation to encompass systemic inequities in service delivery, supply chain reach, and sociocultural acceptance (21, 30). Also, while infrastructural deficits such as the lack of sanitary facilities and waste management are critical, they are compounded by “soft” barriers like cultural taboos and inadequate knowledge. A quasi-experimental study in Cameroon, for instance, demonstrated that a pilot health promotion project focusing on education and community engagement was effective in improving menstrual hygiene practices, even in a context of extreme poverty and entrenched stigma (31). Collectively, these results affirm that strengthening education, subsidising products for the poor, expanding media-based campaigns, and tailoring interventions for rural and formerly married women are essential to reducing menstrual health inequalities. In addressing both the material and structural barriers that limit access to modern products, Ghana can accelerate progress towards Sustainable Development Goal 3, which seeks to ensure healthy lives and promote well-being for all at all ages.

### Implications for public health policy and practice

The findings highlight the need for menstrual health to be integrated into national public health and gender equity frameworks. The Ministry of Health (MoH) and the Ghana Health Service (GHS) should mainstream menstrual health within reproductive, maternal, adolescent, and child health strategies, while Parliamentary Committees on Health and Gender can support the enactment of policies such as tax exemptions or subsidies to make modern menstrual products more affordable. Addressing the socioeconomic and regional disparities observed requires decentralised policy interventions, with Regional Coordinating Councils and Metropolitan, Municipal, and District Assemblies (MMDAs) mandated to develop region-specific action plans that respond to local realities, especially in northern and newly created regions. Education, which was found to be a strong determinant of product use, should be prioritised through the Ministry of Education and the Ghana Education Service by embedding menstrual health into school curricula. Likewise, the Ministry of Sanitation and Water Resources must ensure menstrual hygiene is integrated into WASH policies to guarantee access to safe and private sanitation facilities, while the Ministry of Gender, Children and Social Protection (MoGCSP) should mainstream menstrual equity within gender empowerment programmes. Partnerships with development partners such as UNICEF, UNFPA, and the World Bank, as well as civil society organisations (CSOs), remain critical for technical support, funding, and advocacy to sustain these policy efforts.

Implementation requires strengthening the role of frontline health providers, especially community health nurses and midwives, who should incorporate menstrual health education and product provision into routine services, including adolescent health clinics, antenatal care, and family planning. At the community level, traditional and religious leaders can act as champions to dispel stigma and taboos, while CSOs and local NGOs can lead targeted outreach to underserved groups such as widows, divorced women, and those in poor health. Adolescents, who showed the highest levels of modern product use, can be supported through school health clubs and National Youth Authority programmes that sustain healthy practices into adulthood. Broader public awareness can be advanced through media-driven campaigns, with media houses and telecommunication companies leveraging their reach to promote menstrual equity messages. The private sector, particularly product manufacturers and distributors, should be engaged to expand affordable and sustainable supply chains, with incentives to support local production of low-cost products. By integrating health, education, gender, WASH, and private sector stakeholders, these practices provide a multi-sectoral approach that ensures menstrual products are not only available but also accessible, affordable, and acceptable, ultimately enhancing women's health, dignity, and participation in society.

It should be emphasised that the empirical analyses directly support a focused subset of these actions, namely subsidising or otherwise improving the affordability of products for poorer households, embedding menstrual health within school-based education, expanding mass-media outreach, and strengthening rural WASH infrastructure, each of which corresponds to a determinant identified in the models. The broader multi-sectoral measures outlined above, including taxation, legislative action, and private-sector engagement, are offered as complementary policy directions consistent with the wider literature rather than as direct inferences from the present data, and should be interpreted as hypotheses for further evaluation.

### Strengths and limitations

A major strength of this study lies in its use of the nationally representative data, which provides a large, weighted sample of women of reproductive age and enhances the generalizability of the findings across the country. The analytical approach also strengthens the contribution of this work, as the combination of multilevel regression modelling and non-linear decomposition analysis not only identified individual- and community-level determinants of menstrual product use but also quantified the contribution of compositional and structural factors to observed inequalities. Adding contextual indicators such as community-level education, media exposure, and household wealth, the study was able to capture the influence of broader socio-economic environments on menstrual hygiene practices, offering insights beyond individual-level attributes.

Despite these strengths, certain limitations must be acknowledged. The cross-sectional nature of the DHS restricts the ability to establish causal relationships. Furthermore, reliance on self-reported measures for menstrual product use and related covariates introduces the possibility of recall and social desirability bias. Although the extent of missing data was minimal, the use of listwise deletion may have resulted in some loss of information if excluded respondents differed systematically from those retained. The dataset itself imposes further constraints, as it does not capture important determinants such as the affordability of menstrual products, supply-chain challenges, cultural taboos, or supportive policy environments, which could have enriched the explanatory power of the models. While the models adjusted for multiple covariates, the potential for residual confounding remains, particularly from unmeasured factors such as menstrual pain, stigma, or workplace and school-level support systems. Finally, although regional variations were considered, the models may not fully capture heterogeneity within the newly created administrative regions, where smaller sample sizes could reduce precision in estimates.

In addition, the study relied solely on quantitative survey data; while the models identify factors statistically associated with product use, they cannot capture the underlying mechanisms, meanings, and lived experiences that shape menstrual practices. Complementary qualitative research would therefore be valuable to contextualise these associations and to elucidate the pathways through which socioeconomic and contextual factors operate.

## Conclusions

This study applied non-linear decomposition analysis and predictive multilevel modelling to examine determinants of and inequalities in menstrual product use among Ghanaian women using the 2022 DHS. The findings demonstrate that menstrual product use is strongly stratified by socioeconomic status, education, wealth, and place of residence, with higher uptake among urban, wealthier, and more educated women. Multilevel modelling confirmed significant contextual effects, with 13%–15% of the variance attributable to between-cluster differences, underscoring the role of community-level education, media exposure, and household wealth in shaping menstrual hygiene behaviours. Predictive performance was excellent, with both ordered and binary models achieving strong discrimination.

Decomposition analyses revealed that rural–urban inequalities were driven largely by compositional differences (63% for residence), especially in education and household economic status, while returns to these characteristics also contributed significantly to disparities. These results provide measurable evidence that addressing structural inequities in education, wealth, and community resources could substantially reduce menstrual health gaps. Thus, targeted interventions such as subsidising menstrual products for poorer households, strengthening community education programs, and improving rural WASH infrastructure are achievable and essential strategies to accelerate equitable menstrual health. Given Ghana's policy commitments to universal health coverage and gender equality, scaling such measures within the next 5–10 years would directly contribute to SDG 3 (health), SDG 4 (education), and SDG 5 (gender equality).

## Data Availability

The datasets presented in this study can be found in online repositories. The names of the repository/repositories and accession number(s) can be found below: https://www.dhsprogram.com.
